# Plasma host protein signatures correlating with *Mycobacterium tuberculosis* activity prior to and during antituberculosis treatment

**DOI:** 10.1038/s41598-022-25236-9

**Published:** 2022-11-30

**Authors:** Mame Diarra Bousso Ndiaye, Paulo Ranaivomanana, Lova Tsikiniaina Rasoloharimanana, Voahangy Rasolofo, Rila Ratovoson, Perlinot Herindrainy, Julio Rakotonirina, Matthieu Schoenhals, Jonathan Hoffmann, Niaina Rakotosamimanana

**Affiliations:** 1 Institut Pasteur de Madagascar, Antananarivo, Madagascar; 2 United States Agency for International Development (USAID), Antananarivo, Madagascar; 3 Centre Hospitalier Universitaire de Soins et Santé Publique Analakely (CHUSSPA), Antananarivo, Madagascar; 4 Medical and Scientific Department, Fondation Mérieux, Lyon, France

**Keywords:** Tuberculosis, Infectious-disease diagnostics, Microbiology, Biomarkers, Immunology

## Abstract

There is a need for rapid non-sputum-based tests to identify and treat patients infected with *Mycobacterium tuberculosis* (*Mtb*). The overall objective of this study was to measure and compare the expression of a selected panel of human plasma proteins in patients with active pulmonary tuberculosis (ATB) throughout anti-TB treatment (from baseline to the end of treatment), in Mtb-infected individuals (TBI) and healthy donors (HD) to identify a putative host-protein signature useful for both TB diagnosis and treatment monitoring. A panel of seven human host proteins CLEC3B, SELL, IGFBP3, IP10, CD14, ECM1 and C1Q were measured in the plasma isolated from an HIV-negative prospective cohort of 37 ATB, 24 TBI and 23 HD. The protein signatures were assessed using a Luminex xMAP® to quantify the plasmatic levels in unstimulated blood of the different clinical group as well as the protein levels at baseline and at three timepoints during the 6-months ATB treatment, to compare the plasma protein levels between culture slow and fast converters that may contribute to monitor the TB treatment outcome. Protein signatures were defined using the CombiROC algorithm and multivariate models. The studied plasma host proteins showed different levels between the clinical groups and during the TB treatment. Six of the plasma proteins (CLEC3B, SELL, IGFBP3, IP10, CD14 and C1Q) showed significant differences in normalised median fluorescence intensities when comparing ATB vs HD or TBI groups while ECM1 revealed a significant difference between fast and slow sputum culture converters after 2 months following treatment (*p* = 0.006). The expression of a four-host protein markers (CLEC3B-ECM1-IP10-SELL) was significantly different between ATB from HD or TBI groups (respectively, *p* < 0.05). The expression of the same signature was significantly different between the slow vs the fast sputum culture converters after 2 months of treatment (*p* < 0.05). The results suggest a promising 4 host-plasma marker signature that would be associated with both TB diagnostic and treatment monitoring.

## Introduction

Tuberculosis (TB) is one of the deadliest diseases caused by a single infectious agent as approximately 10 million people are infected each year. As reported by the World Health Organisation (WHO), the mortality rate was 1.5 million from TB in 2020^1^.

The diagnosis of TB is mainly lying on clinical symptoms followed by bacteriological or molecular confirmation of the presence of *Mycobacterium tuberculosis* (Mtb). Once diagnosed the treatment of TB requires antibiotic multitherapies that last at least 6 months and treatment failure as well as relapse can occur^2^. These outcomes are associated with severe adverse effects and long treatment durations that induce a lack of patient adherence to the treatment thus promoting the emergence of drug-resistance^3^. According to the WHO, globally one third of all TB cases are still not notified, and many patients’ samples do not undergo drug-susceptibility testing (DST). Improved TB prevention and control depend critically on the development of a simple, readily accessible rapid test to detect TB and monitor the effect of its treatment. These tests should achieve the WHO Target Product Profile (TPP) recommendations in terms of performances for a non-sputum based screening/triage test (at a sensitivity of > 90%, the minimum specificity as set out in this TPP should be ≥ 70%), for an initial TB diagnostic test to replace sputum based tests (at minimum 60% sensitivity, the minimum specificity should be > 98%) and for a confirmatory test (at minimum 65% sensitivity, the minimum specificity should be > 98%)^4^.

Monitoring TB treatment adherence and effects relies on *Mtb* detection by sputum smear microscopy and culture when possible. Sputum smear microscopy is highly sample- and operator-dependent and has poor sensitivity. The bacteriological confirmation of TB with mycobacterial culture takes from 3 to 6 weeks and it takes longer to obtain the DST results.

On the other hand, molecular tests based on the detection of the mycobacterial DNA like the GeneXpert or the line probe assay showed good specificity/sensitivity and allow rapid identification of antibiotic-resistant *Mtb* strain. However, they may have some limitations, due to the bacterial DNA that can be detected from both live and dead cells.

The development of TB immunodiagnostic tests like the tuberculin skin test (TST) and the interferon gamma release assay (IGRA) offers an alternative to sputum based tests by assessing the peripheral immune response for the identification of individuals infected with *Mtb* but, these tests lack accuracy to monitor treatment. Diagnostic approaches based on non-sputum based tests like the evaluation of host plasma protein, transcriptomic or phenotypic signatures for treatment monitoring, screening or triage showed some relevant clinical advantages^5–11^.

A proteomic study notably described a panel of host protein biomarkers that would help to differentiate active TB from other forms of respiratory disease in non-HIV infected patients^8^. Some of these proteins were particularly described as having potential important roles during the active TB and treatment. The tetranectin, also known as C type LECtin domain family 3 member B (CLEC3B), and extracellular matrix protein 1 (ECM1), are involved in tissue modification and remodeling^12,13^ as well as in pro-/anti-inflammatory and fibrogenic properties and regulating Th2 cell migration^14,15^. The insulin-like growth factor (IGF) pathway 3 (IGFBP3) are regulated in patients with active TB (ATB)^6,8^. SELL is involved in leukocyte addressing, adhesion, migration, signal transduction and has been shown to discriminate TB from other respiratory diseases^16,17^. Soluble CD14 (sCD14) is known for its role in the recognition pathologies in the lungs including active TB^18–22^. C1q, the first subcomponent of the classical complement cascade was used to discriminate ATB from latent TB infection^23–25^. Interferon gamma inducible protein 10 (IP10) is known as a marker for TB infection and was recently described to be involved as an indicator for sputum culture conversion and treatment monitoring^26^. A combination of IP-10 and RANTES has shown good performance in diagnostic and monitoring in pulmonary TB management^27–29^.

The present study aims to compare the expression of these proteins previously described as plasma host markers related to TB, CLEC3B, SELL, IGFBP3, IP10, CD14, ECM1 and C1Q, in different human clinical groups (ATB, TBI, and HD) and during anti-TB treatment to identify a putative host protein signature useful for both TB diagnosis and treatment monitoring.

## Results

### Sociodemographic and clinical characteristics

A total of 37 patients with bacteriologically confirmed pulmonary tuberculosis (ATB), 23 individuals with asymptomatic tuberculosis infection (TBI), and 24 healthy donors (HD) were enrolled in the study. The sociodemographic and clinical characteristics of the participants at baseline are summarized in Table 1. Among patients with ATB, 16.2% reported previous TB infection. All the 37 TB patients successfully achieved their TB treatment. No drug resistance was reported amongst the ATB group. At baseline, 32.4% (12/37) of the ATB cases were reported as negative by smear microscopy, while 40.5% (15/37) were high grade (2+ or 3+) positive smear and 27% (10/37) were low grade (1+ or scanty) positive smear.

**Table 1:** Sociodemographic data of patients.

	ATB	TBI	HD	*p* value
	N = 37	N = 23	N = 24	
**Patient demographics**				
Age (years)	28 (22–43)	35 (24.5–44.5)	29.5 (23.25–36.25)	0.22
Sex (male)	59.5% (22/37)	34.8% (8/23)	20.8% (5/24)	0.015
BMI at inclusion	17.27 (16.16–18.48)	NA	NA	
**Risk factors and comorbidities**				
Smoking	40.5% (15/37)	NA	NA	
Alcohol abuse	40.5% (15/37)	NA	NA	
Jail detention history	2.7% (1/37)	NA	NA	
Chronic HCV infection	2.7% (1/37)	NA	NA	
**History of TB**				
Previous TB	16.2% (6/37)	NA	NA	
**Previous TB treatment outcome**				
Cured and completed	50% (3/6)	NA	NA	
Treatment failure	16.7% (1/6)	NA	NA	
Outcome not evaluated or unknown	33.3% (2/6)	NA	NA	
**TB characteristics at inclusion**				
Drug-susceptible Mtb	100% (37/37)	NA	NA	
Pulmonary TB	100% (37/37)	NA	NA	
**Sputum smear microscopy at inclusion**				
High grade (2+ or 3+)	40.5% (15/37)	NA	NA	
Low grade (1+ or scanty)	27% (10/37)	NA	NA	
Negative	32.4% (12/37)	NA	NA	
**Treatment regimen**				
2HRZE/2HR	97.3% (36/37)	NA	NA	
Slow converters	32.4% (12/37)	NA	NA	
Fast converters	67.6% (25/37)	NA	NA	
WBC absolute count at inclusion (/mm^3^)	8870 (6350–11,360)	7340 (5875–9230)	6760 (5920–7132.5)	0.009
Lymphocyte at inclusion (% of WBC)	17.8 (13–23.9)	35.2 (30.1–41.4)	43.1 (38.25–52.67)	< 0.001
Monocytes at inclusion (% of WBC)	9.8 (7–11.5)	7.2 (6.6–7.75)	7.65 (6.8–9.47)	0.001
Hemoglobin at inclusion (g/dL)	11.9 (11–13.1)	14.4 (13.6–15.35)	14.35 (13–15.1)	< 0.001
Neutrophils at inclusion (% of WBC)	70.1 (62.7–77.5)	52 (45.7–58.56)	44.6 (33.57–48.82)	< 0.001
Eosinophil at inclusion (% of WBC)	1 (0.5–1.9)	2.6 (1.91–4.99)	2.71 (2.18–4.28)	< 0.001
Basophils at inclusion (% of WBC)	0.4 (0.1–0.6)	0.6 (0.5–0.85)	0.75 (0.5–0.89)	< 0.001
BCG vaccination	91.9% (34/37)	NA	NA	
Positive QuantiFERON-TB gold plus at baseline	67.6% (25/37)	100% (23/23)	0% (0/24)	

N(IQR); %(n/N).

The white blood cell count (WBC) was higher in patients with ATB compared to respectively TBI or HD (8870/mm^3^ (6350–11,360), 7340/mm^3^ (5875–9230) and 6760/mm^3^ (5920–7132.5) respectively, *p* < 0.001). In contrast, the proportion of lymphocytes in the WBC count was lower in ATB group than in TBI and HD groups (17.8% [95% CI 13–23.9], 35.2% [95% CI 30.1–41.4] and 43.1% [95% CI 38.25–52.67] respectively, *p* < 0.001). Retrospectively “Slow converter” profile was assigned to 32.4% (12/37) of ATB patients. QFT-P assay result was positive for 67.6% (25/37) of ATB cases at baseline.

### Evaluation of single host protein markers related to clinical group and mycobacterial load variations

**Figure 1:**
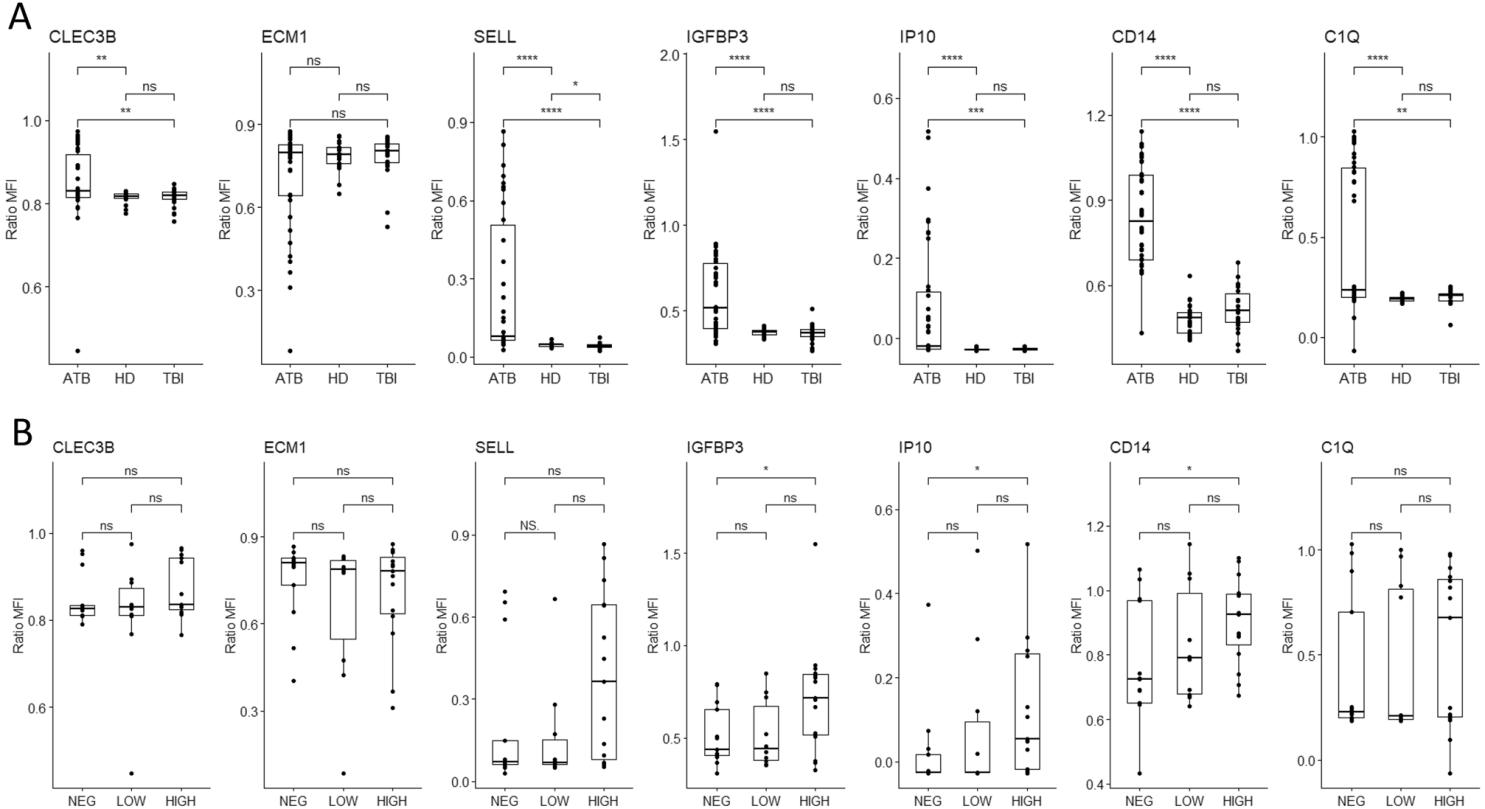
Evaluation of single host protein markers related to clinical group and mycobacterial load variations. (**A**) Comparison of markers expression in different clinical groups. (**B**) Comparison of markers expression and mycobacterial load variation. Data are given as median + interquartile range. Each black dot represents one patient. Data were compared using the Mann–Whithney U test with correction apply to adjust *p* values. *p < 0.05; **p < 0.01; ***p < 0.001.

The differences in normalized MFI ratio of each marker were separately assessed and compared between the three studied clinical groups as well as during the ATB treatment (Fig. 1). When comparing the protein levels in ATB vs HD or in ATB vs TBI, significant differences of normalized MFI ratio were observed for all markers except for ECM1 (*p* > 0.05) (Fig. 1A).

**Figure 2:**
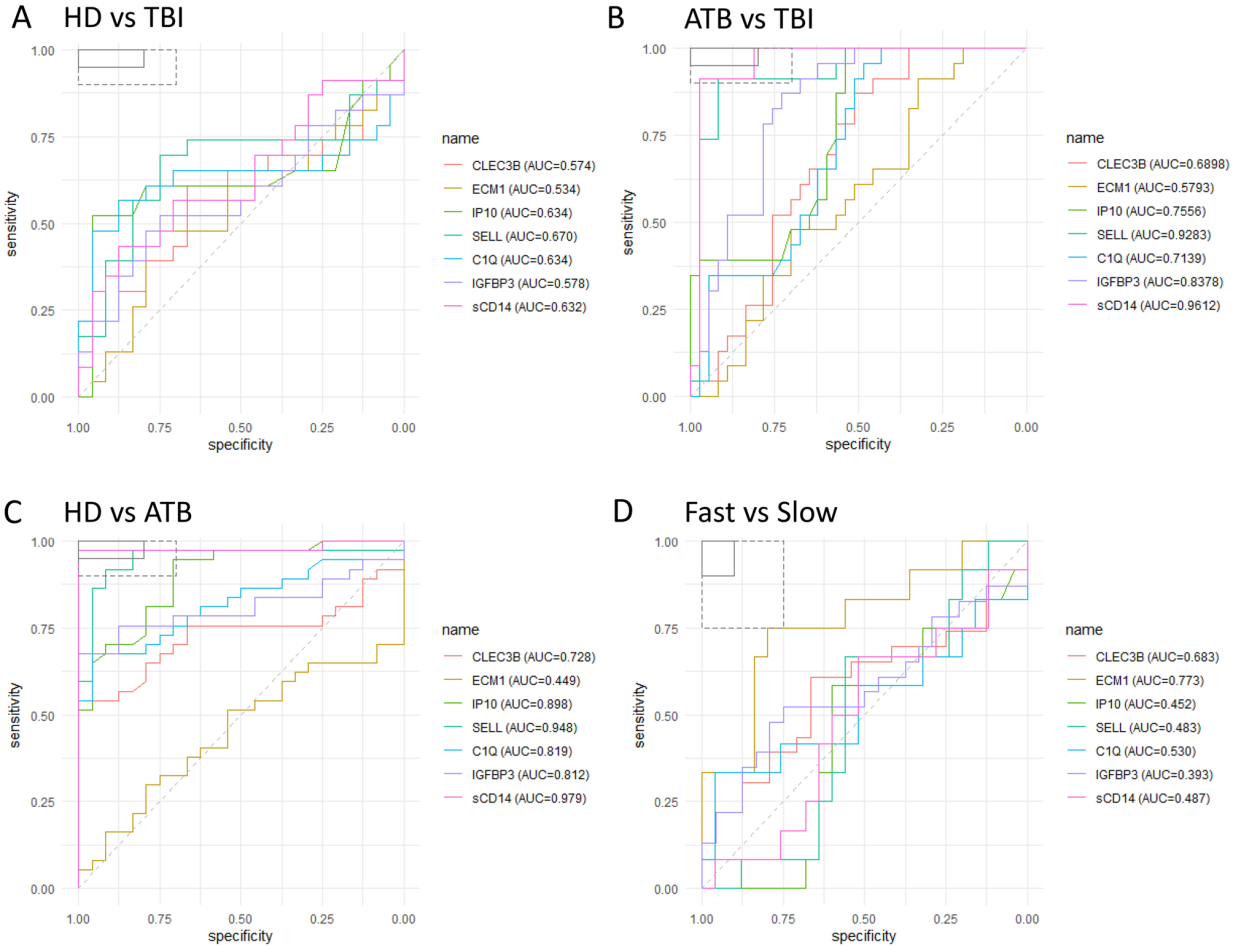
Receiver operating characteristic (ROC) curve analysis of biomarkers for triage and TB treatment. ROC curves for comparing the performance of different markers between healthy donors (HD) and TB infected (TBI) (**A**), active TB infection (ATB) vs. TBI (**B**), HD vs. ATB (**C**), and finally Fast and Slow treatment responders, are shown. In the top left box, the solid and dashed lines represent the respective optimal and minimum criteria set by the WHO in the target product profile (TPP) for a triage test for TB.

A significant difference in normalized MFI ratios was observed for SELL levels when comparing TBI to HD (*p* = 0.046). The use of plasma measure of sCD14 and SELL to distinguish ATB from HD reached respective sensitivity of 97% [95% CI 85–99] and specificity of 96% [95% CI 80–10] for sCD14 and a sensitivity of 97% [95% CI 86–99], specificity of 100% [95% CI 85–100] for SELL (Fig. 2C). sCD14 and SELL distinguished also TBI from ATB (Fig. 2B). Simplex detection of these 2 markers did not discriminate TBI from HD (Fig. 2A).

Due to the various sputum smear microscopy observed at baseline for the ATB patients that may influence the immune response and the plasma protein levels, we wondered if the expression of these markers and the mycobacterial loads were correlated. The levels of the plasma proteins were thus stratified to the sputum smear microscopy grades at baseline we observed for the ATB (Fig. 1B). Among the seven markers, IGFBP3, IP10, and sCD14, had a significant difference of normalized MFI ratio between negative and high-grade positive smears: *p* = 0.029, 0.030, and 0.037 respectively. No significant difference was noted when comparing the expression of those 3 host proteins between patients with low- vs high-grade nor between negative smear grade vs low-grade (Fig. 1B).

### ECM1 plasma levels differ according to the sputum culture conversion

**Figure 3:**
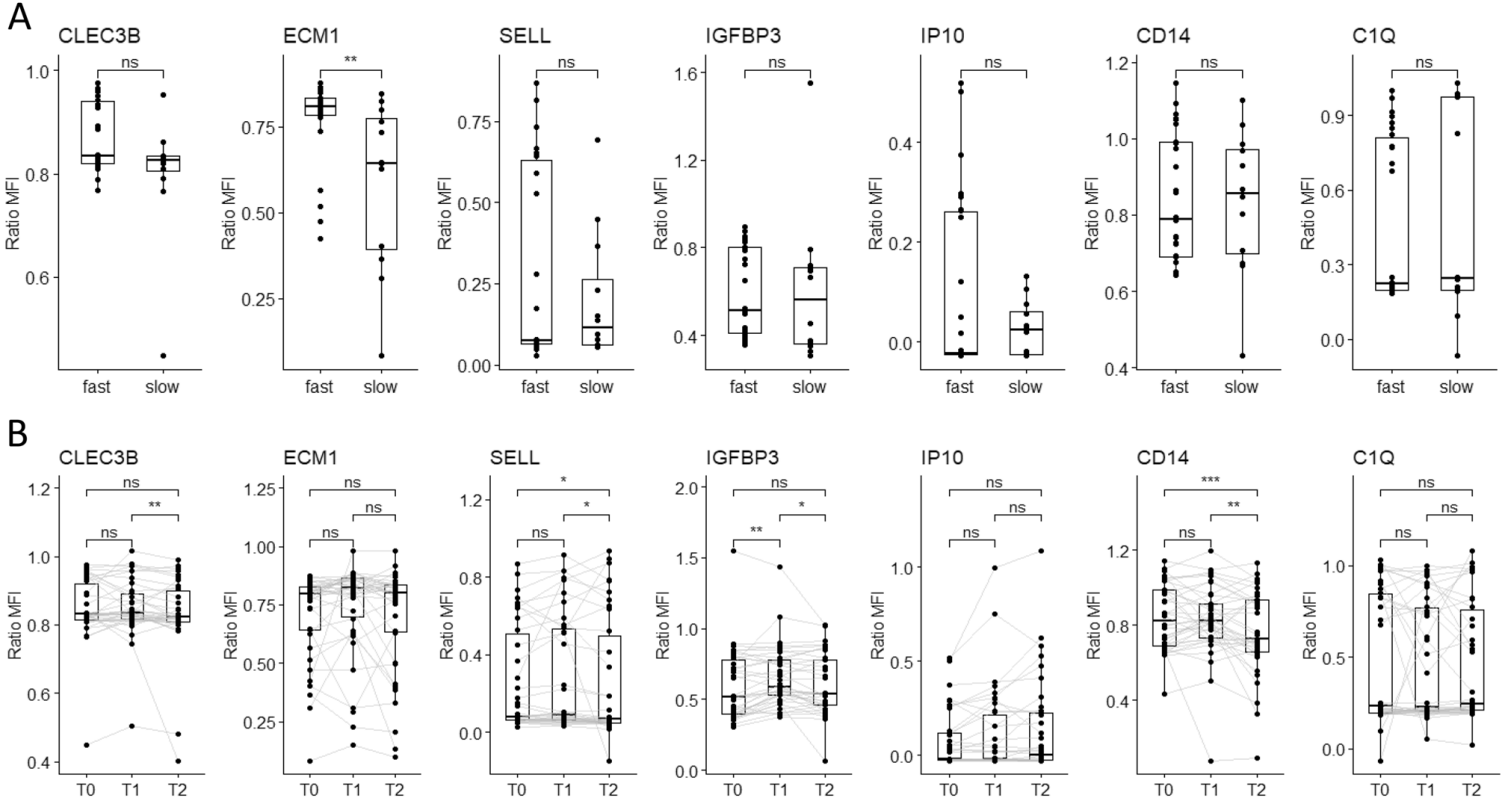
Plasma markers levels for treatment monitoring. (**A**) Comparison of markers expression in Fast vs Slow. (**B**) Dynamics of plasma markers over the course of TB therapy (n = 37 per timepoint). Data are given as median with interquartile range. Each black dot represents one patient, Grey lines connect data points from the same patient T0: baseline. T1: baseline + 2 months. T2: end of treatment. Data were compared using the Wilcoxon–paired test or Mann-Whithney U test with correction apply to adjust *p* values. *p < 0.05; **p < 0.01; ***p < 0.001.

Regarding the treatment monitoring, all the 37 ATB patients had achieved sputum conversion at the end of treatment (T2). A significantly higher (*p* = 0.006) level of ECM1 normalized MFI at baseline was observed in patients with fast culture conversion status compared to those of the slow converters (Fig. 3). ROC curve analysis of plasma ECM1 levels distinguished the two clinical groups between "fast converters" and "slow converters" at baseline with an AUC of 0.773, sensitivity of 75% [95% CI 47–91], and specificity of 80% [95% CI 61–91] (Fig. 2D).

### Identification and evaluation of a four host plasma protein signature between the clinical groups

A CombiROC algorithm was used to identify the best plasma marker combinations that first allowed to distinguish the three clinical groups (ATB, TBI, and HD). A set of 120 signatures were obtained from the seven studied markers (Supplementary tables 2–8). These signatures were ranked according to their decreasing Area Under the Receiver Operating characteristic Curve (AUC) values, then, the number and the relevance of the combined markers involved in each signature. The “ECM1-CLEC3B-IP10-SELL” combination was the most relevant to stratify the clinical groups regarding the selected parameters. This protein combination reached an AUC of 0.95, corresponding to a sensitivity of 95% and specificity of 92% when comparing the ATB protein levels to those of the HD group (Table 2). This same signature also showed an AUC of 0.87 for a treatment monitoring assay, corresponding to a sensitivity of 83%, and specificity of 84% in identifying fast or slow converters at baseline prior to TB treatment.

**Table 2:** Performance of CLEC3B-ECM1-IP10-SELL signature for comparison of clinical groups and TB treatment monitoring.

Purpose	Groups	AUC	SE	SP	CutOff	ACC	TN	TP	FN	FP	NPV	PPV
**Clinical group comparisons**												
	ATB vs HD	0.958	0.946	0.917	0.412	0.934	22	35	2	2	0.917	0.946
	ATB vs TBI	0.929	0.892	0.913	0.536	0.9	21	33	4	2	0.84	0.943
	HD vs TBI	0.741	0.565	0.958	0.564	0.766	23	13	10	1	0.697	0.929
**Treatment monitoring**												
	Fast vs Slow converters	0.87	0.833	0.84	0.272	0.838	21	10	2	4	0.913	0.714

*AUC* area under the ROC curve, *SE* sensitivity, *SP* specificity, *PPV* positive predictive value, *NPV* negative predictive value, *ACC* accuracy.

For the discrimination between ATB and TBI individuals, the AUC value was 0.929 corresponding to a sensitivity of 89% and specificity of 91%.

After comparing the protein levels observed from TBI to those of HD, a lower performance was observed compared to the latest groups with an AUC value of 0.74 corresponding to a sensitivity of 56% and specificity of 96%.

## Discussion

The plasma host expression variations of seven proteins in patients with ATB (with different time-points from baseline to the end of anti-TB treatment), subjects with *Mtb* infection and healthy donors has been assessed in this study. Among the seven proteins targeted, our results suggest that a signature of four plasma proteins seems useful for both TB diagnostic and treatment monitoring. Nevertheless, its diagnostic/prognostic performance must be confirmed in a large-scale clinical study. To date, few studies have demonstrated the existence of a unique signature fulfilling the WHO TPPs recommendations for both purposes^4^.

Several studies have already described marker signatures of interest for TB triage^28–31^. Chegou et al., identified signatures on QFT supernatants using the same technology (i.e. Luminex xMAP® technology) for TB diagnosis. A biosignature including IFN-γ, MIP-1β, TGF-α in unstimulated plasma, and antigen-specific TGF-α and VEGF has been described with acceptable AUC of 0.81 to discriminate between group of patients with TB disease or other respiratory diseases (ORD)^32^. In another study, a five-marker (IL-1β, IL-23, ECM1, HCC1, fibrinogen) biosignature was identified in saliva for TB diagnosis with an optimal AUC of 0.88^33^. In both studies, TPPs recommendations for a triage test were not reached, and the utility of these signatures in treatment monitoring was not evaluated.

In the present study, the protein markers expression in the plasma were assessed using a multiplex assay developed on the xMAP platform and were then analysed individually or in combined panels to establish a signature associating both TB detection and treatment monitoring. The four host-plasma marker signatures (ECM1-CLEC3B-IP10-SELL) selected in our study would meet the recommendations for a non-sputum-based assay, however, it needs to be evaluated in a larger scale sample size study population, allowing to better define the diagnostic/prognostic performance of this assay.

Regarding the host proteins detected with our xMAP panel, ECM1 has already been described as a potential TB treatment monitoring marker^34^. This marker, along with other proteins, can distinguish fast from slow responders in sample comparisons at baseline and 8 weeks after TB treatment, using the a complex multiplexed, aptamer-based proteomic technology, SOMAscan^34^. In our study, we demonstrate that the level of ECM1 changes significantly prior to TB treatment and the issue of the sputum culture conversion after 2 months of therapy. This host marker might be of interest to identify at baseline patients who would require close follow-up during the intensive phase of treatment. The use of this type of marker could also help refine therapeutic trials aimed at shortening treatment or evaluating shorter TB treatment regimens.

After combining different plasma proteins, we showed that the combination of ECM1, CLEC3B, IP10 and SELL generated the best AUC to discriminate (1) ATB from HD groups (95% sensitivity and 92% specificity), and (2) fast vs slow sputum culture converters at baseline (83%sensitivity and of 84% specificity). If the diagnostic/prognostic performance of this four host-plasma marker signature (ECM1-CLEC3B-IP10-SELL) are confirmed to meet the TPP recommendations for both purposes, this potential signature will present several assets : its detection can directly be measured from unstimulated plasma (as already described elsewhere^27,28,35^) or directly assessed for instance on a xMAP® luminex platform from which results interpretation is not biased by the analytes concentrations determination. On this latter point, it has been shown that normalized MFI ratios are generally a better choice than absolute concentration values for statistical analysis as it does not require background subtraction for differentialanalysis^36,37^. Host biomarkers detection from unstimulated plasma might be of interest for the diagnosis of paucibacillary forms of TB (i.e., childhood TB and/or extra-pulmonary TB).

The present study has limitations. The evaluation was only carried out on a limited sample size of patient cohorts that do not allow to powerfully assess the diagnostic value of these proteins as TB biomarkers. The efficacy of the treatment such as the success or the treatment failure cannot be evaluated in this study, as none of the patients had a treatment failure nor drug-resistance profile after the 6-months treatment period. These results need to be validated in larger scale studies using diverse endemic and genetically different populations to further appreciate the robustness of the biosignatures.

In conclusion, the present study identified four host-plasma proteins marker that can potentially be useful as biological markers for both TB diagnostic and treatment monitoring. The diagnostic and prognostic performances of these protein markers must be confirmed in larger clinical studies. Implementing such protein markers or biosignatures in limited resource countries and/or those countries with the highest TB incidences could help to improve the diagnosis and the global management of TB.

## Materials and methods

### Study design and population

All the participants were recruited in Antananarivo, Madagascar. Between January to April 2019, active TB patients (ATB) were enrolled from the individuals presenting TB symptoms addressed for TB diagnosis at the main anti-tuberculosis centre of Madagascar at the Centre Hospitalier Universitaire de Soins et Santé Publique in Analakely. Inclusion criteria for ATB are pulmonary TB diagnosed patients adult, ≥ 18 years old patients identified using both bacteriological and/or molecular tests, ie. scoring positive for pulmonary TB with Mtb detection either by sputum smear microscopy and/or by Xpert MTB/RIF Ultra and/or sputum culture on Lowenstein-Jensen (LJ) media.

Healthy volunteer donors (HD), recruited as a control group were clinically asymptomatic adults (≥ 18 years old) without any sign of TB and without known TB contact. For the study, QuantiFERON-TB Gold (QFT-P) plus was performed on the healthy volunteers and those positive to QFT-P (ie. IFN-γ production ≥ 0.35 IU/mL in response to TB1 and TB2 stimulation) were classified in the TBI group. Pregnant women, HIV-positive individuals, people living with known diabetes mellitus comorbidity and patients under immunocompromising treatment were excluded from this study.

### Mycobacteriological procedures

ATB diagnosis was based on both bacteriological and molecular tests. At least one sputum sample was collected at inclusion (T0) for Xpert MTB/RIF Ultra (Cepheid) or culture testing on solid culture Lowenstein–Jensen media and tested by smear microscopy for the presence of acid-fast bacilli (AFB) using the Ziehl–Neelsen staining method and/or Auramine O staining. Smear microscopy results were classified as negative smear, low-grade positive smear (1+ or scanty) and high-grade positive smear (2+ or 3+). Confirmed TB patients were re-evaluated by sputum smear and culture during the intensive phase of their treatment (T1) thereafter at the end of treatment (T2) and 2-months after treatment completion (T3) to confirm that they were successfully treated and cured. Drug susceptibility testing (DST) methods were performed according to standard protocols^38^.

### TB treatment and follow-up

Confirmed TB patients were put on Directly Observed Treatment Strategy and received a 6 months treatment with four antibiotics Rifampicin (R), Isoniazid (H), Ethambutol (E) and Pyrazinmide (Z) according to Madagascar standard protocols (2EHRZ/4RH)^39^. During their treatment, TB patients were followed up at inclusion (T0), after 2 months of treatment (T1), at the end of therapy (T2); 6 months for drug susceptible patients. Sputum culture conversion at T1 was used to define three patient subsets: fast converters (definitive culture conversion between T0 and T1), slow converters (definitive culture conversion between T1 and T2), and patients with poor treatment outcome (positive culture results at T2: treatment failure; or positive culture at T3: relapse) (Supplementary figure 1).

### Blood collection process

A minimum of 6 mL of peripheral whole blood were collected from each participant: 1 mL was collected in EDTA tubes for whole blood cell counting with the Sysmex XT-2100i haematology cell counter according to manufacturer instructions, and 5 mL were drawn in Lithium heparin tubes for IGRA.

For QFT-P assay, 1 mL of whole blood was collected directly into each of the four QFT-P tubes (Qiagen, Hilden, Germany, 622526) provided by the QFT-P kit (Nil: Negative Control, TB-Antigens (TB1/TB2) and Mitogen: Positive control). After 16–24 h incubation at 37 °C, plasma samples were harvested and stored at − 80 °C prior to measures using QFT-P ELISA Kit (Qiagen, Hilden, Germany, 622120), according to the manufacturer instructions. Briefly, 50 µL of plasma samples were used and optical density (OD) results were compared to log-normalized values from a freshly reconstituted IFN-γ standard kit. To consider any potential immunomodulation phenomena unrelated with TB treatment, baseline IFN-γ level values (Nil tubes) were subtracted from antigen-stimulated IFN-γ values (TB1, TB2 and Mitogen). According to the kit’s sensitivity range, the maximum for IFN-γ level values was set at 10 IU/mL and negative values were rescaled to zero.

### Luminex xMAP® assay set-up

In the framework of this study a multiplex detection panel of CLEC3B, SELL, IGFBP3, IP10, CD14, ECM1 and C1Q in plasma has been set-up using the Luminex xMAP® technology (Table 3). Coupling of antibodies to beads was performed according to the manufacturer’s instruction43. All antibodies, recombinant proteins and bead regions used in this study are listed in supplementary table 1. After coupling confirmation, reaction parameters including the capture antibody concentration, the detection of antibody concentration, and the number of washing steps were tested to optimize the assay protocol. The optimal assay protocol generated a mixture of each antibody-coupled microsphere that was diluted in an assay buffer to 50 beads of each region per µL. 50 µL of bead suspensions and 50 µL of assay buffer were pipetted into each well. Standard curves were obtained using a tenfold dilution in an assay buffer of recombinant proteins (10000 ng/mL to 0.01 ng/mL) that were also used as positive controls. Each 96 well plate received 50 µL of standard, plasma or assay buffer only (blanks), bringing the final volume to 150 µL per well. Plates were incubated for 30 min on a plate shaker, then washed with an assay buffer. A mixture of biotinylated detection antibodies (4 µg/mL) was added to each well and incubated for 30 min. The beads were washed and incubated with PE-labelled streptavidin SAPE (diluted to 4 µg/mL in assay buffer) for 30 min. The beads were washed and then resuspended in a 100 µL assay buffer before analysis on the MAGPIX Luminex platform. All incubations were performed in the dark, at room temperature on a shaker. To normalize the data and eliminate interpolation bias, median fluorescence intensity (MFI) ratios were evaluated as follow:

$$Normalized\,MFI\,ratio = \frac{MFI\,x - MFI\,min}{{MFI\,max - MFI\,min}}$$

**Table 3:** List of host markers evaluated in this study.

Markers	Full name	Function
CLEC3B	C-type lectin domain family 3 member B/Tetranectin	Transport/tissue remodelling^47^
ECM1	Extracellular Matrix Protein 1	Tissue development and remodelling^48^
sCD14	Monocyte differentiation antigen sCD14 soluble	Immune response^49^
SELL	Selectin L/CD26L	Cell migration and adhesion^50^
IGFBP3	Insulin-like growth factor-binding protein 3	Cell proliferation^51^
C1q	Complement component	Complement^52^
IP10	Interferon gamma-induced protein 10/CXCL10	Immune response^53^

The MFI min and max correspond to the MFI values of the protein given at the concentration 0.01 ng/mL (min) and 1000 ng/mL (max). X represents the sample.

### Statistical analysis

At enrolment and at each follow-up visit for the TB patients, medical history, pseudonymized clinical and demographic data were collected using standardized questionnaires and stored in a secured cloud-based database system (CASTOR Electronic Data Capture, Version 1.4, Netherlands)^40^.

Data analyses were performed using R software version 4.0.3^41^. Due to the studied sample size, discrete variables were analysed using Fisher’s Exact test with Bonferroni’s post-hoc correction test^42^. Normality was assessed using the Shapiro–Wilk Normality test. Normal, continuous variables were analyzed with Student’s t-test. Non-normal, continuous variables were analyzed with the Mann–Whitney test or the Kruskal–Wallis rank-sum test with Dunn's Kruskal–Wallis Multiple Comparisons post-hoc test^43^. Repeated measures of non-independent continuous variables were analyzed using the Friedman rank-sum test, with Wilcoxon-Nemenyi-McDonald-Thompson’s post-hoc test^44^. For both ROC analyses and logistic regression, model performance metrics (respectively, AUC and the C-statistic) were corrected for optimism using bootstrap to assess model validity as described elsewhere^45^. Combinatorial analysis of multiple biomarkers to define the best marker combinations of the tested plasma markers was done using the CombiROC package^46^. The combinations with the highest AUC, sensitivity and specificity values were considered for selection of efficient immune biomarker signatures. Computation and selection of optimal biomarker combinations by integrative ROC.

### Ethics statement

The study protocol was reviewed and approved by the Ministry of Public Health and the Ethical Committee for Biomedical Research of Madagascar (Reference number: n°099–MSANP/CERBM). All study participants provided written informed consent. All research was performed in accordance with relevant guidelines and regulations.

## Supplementary Information

Supplementary Information 1.

Supplementary Information 2.

## Data Availability

All raw data will be shared upon request to the corresponding author.
