# Supplementary material for: Plasma host protein signatures correlating with Mycobacterium tuberculosis activity prior to and during antituberculosis treatment
Source: Sci Rep. 2022 Nov 30;12:20640. doi: 10.1038/s41598-022-25236-9 (PMC9712643; doi:10.1038/s41598-022-25236-9)
Supplement: Supplementary file 2 — Supplementary Information 2. [file 41598_2022_25236_MOESM2_ESM.pdf]

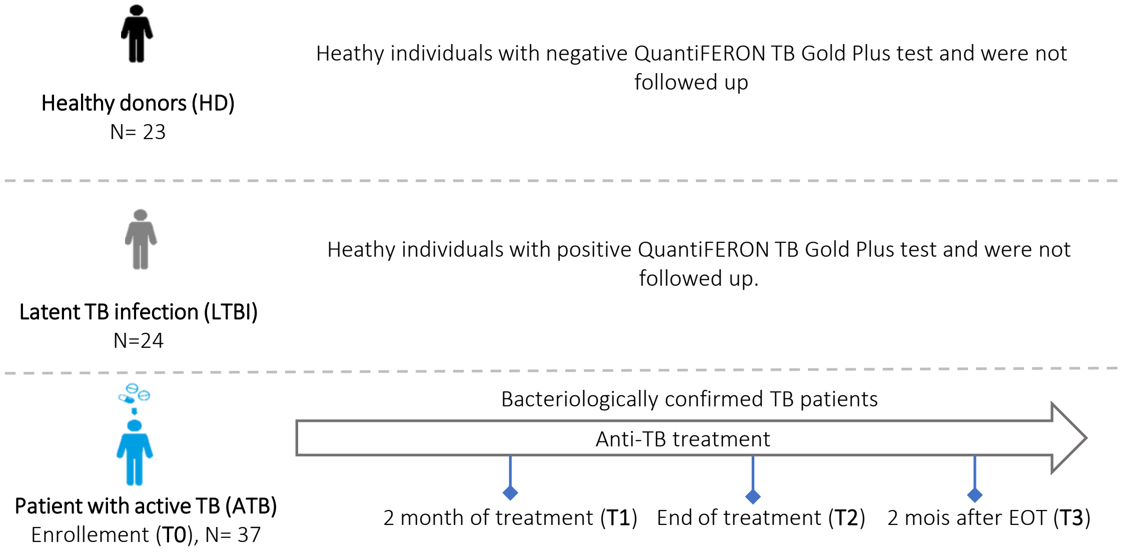

	Culture		
	T0	T1	T2
Fast converters (N=25)	+	-	-
Slow converters (N=12)	+	+	-

Fast converters: culture conversion between T0 and T1
Slow converters : culture conversion between T1 and T2

Supplemantary Figure 1. Flow diagram describing the enrollment and exclusion of participants with active TB, latent TB infection, and healthy donor participants from the different cohorts. ATB patients were followed-up at four different time points throughout antibiotic therapy: at month 2 (T1), at the end of treatment (T2), and 2 months after treatment completion (T3). Fast converters are ATB patients with culture conversion between T0 and T1. Slow converters are ATB patients with culture conversion between T1 and T2
